# Total daily energy expenditure is increased following a single bout of sprint interval training

**DOI:** 10.1002/phy2.131

**Published:** 2013-10-24

**Authors:** Kyle J Sevits, Edward L Melanson, Tracy Swibas, Scott E Binns, Anna L Klochak, Mark C Lonac, Garrett L Peltonen, Rebecca L Scalzo, Melani M Schweder, Amy M Smith, Lacey M Wood, Christopher L Melby, Christopher Bell

**Affiliations:** 1 Department of Food Science and Human Nutrition, Colorado State UniversityFort Collins, Colorado; 2 Division of Endocrinology Metabolism and Diabetes, University of Colorado Anschutz Medical CampusDenver, Colorado; 3 Division of Geriatrics, University of Colorado Anschutz Medical CampusDenver, Colorado; 4 Department of Health and Exercise Science, Colorado State UniversityFort Collins, Colorado

**Keywords:** Exercise, metabolism, thermogenic, weight maintenance

## Abstract

Regular endurance exercise is an effective strategy for healthy weight maintenance, mediated via increased total daily energy expenditure (TDEE), and possibly an increase in resting metabolic rate (RMR: the single largest component of TDEE). Sprint interval training (SIT) is a low-volume alternative to endurance exercise; however, the utility of SIT for healthy weight maintenance is less clear. In this regard, it is feasible that SIT may evoke a thermogenic response above and beyond the estimates required for prevention of weight gain (i.e., >200–600 kJ). The purpose of these studies was to investigate the hypotheses that a single bout of SIT would increase RMR and/or TDEE. Study 1: RMR (ventilated hood) was determined on four separate occasions in 15 healthy men. Measurements were performed over two pairs of consecutive mornings; each pair was separated by 7 days. Immediately following either the first or third RMR measurement (randomly assigned) subjects completed a single bout of SIT (cycle ergometer exercise). RMR was unaffected by a single bout of SIT (7195 ± 285 kJ/day vs. 7147 ± 222, 7149 ± 246 and 6987 ± 245 kJ/day (mean ± SE); *P* = 0.12). Study 2: TDEE (whole-room calorimeter) was measured in 12 healthy men, on two consecutive days, one of which began with a single bout of SIT (random order). Sprint exercise increased TDEE in every research participant (9169 ± 243 vs. 10,111 ± 260 kJ/day; *P* < 0.0001); the magnitude of increase was 946 ± 62 kJ/day (∼10%). These data provide support for SIT as a strategy for increasing TDEE, and may have implications for healthy body weight maintenance.

## Introduction

In light of the disappointing results of efforts to curb the global obesity epidemic, several attempts have been made to calculate the minimum increase in total daily energy expenditure required to prevent adult weight gain. Assuming no change in dietary intake, based on metabolic efficiency and the caloric cost of weight gain (fat deposition), an increase in total daily energy expenditure of approximately 200–600 kJ (∼50–150 kcal) has been estimated as sufficient to prevent weight gain in ∼90% of adults living in industrialized countries (Hill et al. 2003; Stroebele et al. 2011). Accordingly, multiple strategies have been investigated that might evoke such an increase. These have included pharmacological interventions (e.g., ephedrine [Diepvens et al. 2007; Dulloo et al. 1992]), thermogenic beverages and/or supplements (e.g., green tea/green tea extracts [Auvichayapat et al. 2008; Lonac et al. 2010]), and manipulation of environmental conditions (such as room temperature [Ravussin et al. 2012]). Arguably, the most reliable method of increasing total daily energy expenditure, and the intervention that has the most favorable “side-effects”, is exercise. Unfortunately, long-term compliance with a program of traditional endurance exercise is notoriously poor, and the associated perceived time commitment is most commonly cited as the obstacle (Stutts 2002). Sprint interval training (SIT) is now widely recognized as a time-efficient, low-volume alternative to endurance exercise training. Multiple studies have identified important and clinically relevant physiological adaptations to SIT; these include both metabolic and cardiovascular benefits (Rakobowchuk et al. 2008; Babraj et al. 2009; Little et al. 2010; Richards et al. 2010a; Whyte et al. 2010; Gillen et al. 2012; Cocks et al. 2013; Shepherd et al. 2013). The utility of SIT for prevention of weight gain and/or weight loss is less clear. In this regard, it is feasible that SIT may evoke a thermogenic response above and beyond the estimates required for prevention of weight gain.

This manuscript details two studies examining the thermogenic response to a single bout of SIT. In study 1, the influence of SIT on resting metabolic rate (RMR; the single largest component of total daily energy expenditure) was determined. As a secondary aim, the response to SIT of circulating factors known to be of thermogenic/metabolic importance was also studied. In study 2, the influence of a single bout of SIT on total daily energy expenditure was quantified (via whole-room indirect calorimetry), and as a secondary aim, total daily fat oxidation in response to SIT was also examined. We hypothesized that a single bout of SIT would: (1) increase RMR measured the following day; and (2) increase total daily energy expenditure during the day the bout of SIT was undertaken. An increase in energy expenditure greater than 200–600 kJ would provide support for SIT as strategy for weight maintenance.

## Methods

### Subjects – studies 1 and 2

We studied 27 adult males (15 in study 1 and 12 in study 2). None of the participants from the first study participated in the second study. Selected physical characteristics from research participants are presented in Table 1. Inclusion criteria for both studies consisted of a recreationally active lifestyle (that is, habitual exerciser but not competitive beyond the intramural level), normal fasting blood glucose concentration (< 5.5 mmol/L [<100 mg/dL]), and normal blood pressure (<140/90 mmHg). Exclusion criteria included regular use of tobacco products or medications that might confound the interpretation of data, and contraindications to vigorous exercise (as determined by 12-lead beat-by-beat electrocardiogram and blood pressure measurements at rest and during incremental exercise). Consequently, research participants demonstrated physiological attributes typical of healthy young men. That is, on average they were of normal weight (based on body mass index and body composition), of average aerobic capacity (based on peak oxygen uptake [VO_2peak_]), and free of cardiovascular and metabolic disease. The experimental protocol conformed to the standards set by the Declaration of Helsinki of 1975, as revised in 1983, and was approved by the Institutional Review Board at Colorado State University (Studies 1 and 2) and the Colorado Multiple Institutional Review Board (study 2). The nature, purpose, and risks of the study were explained to each research participant before written informed consent was obtained.

**Table 1:** Selected research participant characteristics from study 1 and study 2.

	Study 1	Study 2
*n* (all male)	15	12
Age (years)	23 ± 1	26 ± 2
Body mass (kg)	78.7 ± 2.6	73.5 ± 2.2
Height (m)	1.81 ± 0.2	1.76 ± 0.2
BMI (kg/m^2^)	24.4 ± 0.8	23.6 ± 0.5
% Body fat	17.1 ± 1.4	17.2 ± 0.8
Fat mass (kg)	13.6 ± 1.4	12.8 ± 0.9
Fat-free mass (kg)	64.0 ± 1.8	59.7 ± 1.5
VO_2peak_ (mL kg^−1^ min^−1^)	47.5 ± 1.7	53.0 ± 2.0
HR_peak_ (beats/min)	193 ± 2	181 ± 3
RER_peak_	1.14 ± 0.02	1.13 ± 0.02

Data are mean ± SE. BMI, body mass index; VO_2peak_, peak oxygen consumption; HR_peak_, peak heart rate; RER_peak_, peak respiratory exchange ratio.

### Procedures common to both studies – screening

Fat mass and fat-free mass were measured using dual-energy X-ray absorptiometry (DXA-IQ; Lunar Radiation corp., Madison, WI, software version 4.1). VO_2peak_ was determined with a metabolic cart (Parvo Medics, Sandy, UT) during incremental cycle ergometer exercise (25–30 W/min) to volitional fatigue, as previously described (Richards et al. 2010a,b). Prior to and during exercise, beat-by-beat heart rate was assessed via 12-lead electrocardiography; electrocardiograms were reviewed by a cardiologist prior to further study participation.

### Study 1 – influence of SIT on RMR

Following screening, research participants (*n* = 15) reported to the laboratory on four separate occasions for the measurement of RMR. Measurements were performed over two pairs of consecutive mornings; each pair was separated by 7 days. RMR was measured over 45 min, as previously described (Newsom et al. 2008, 2010). The first 15 min was considered a habituation period and was excluded from final analysis. VO_2_ and carbon dioxide production (VCO_2_) were averaged each minute for 30 min using a custom-built, ventilated hood, indirect calorimetry system (Nighthawk Design, Boulder, CO). The system was calibrated daily with precision mixed gases (Airgas, Denver, CO). Energy expenditure was calculated using the Weir formula (Weir 1949).

Immediately following either the first or third RMR measurement (randomly assigned) research participants completed a single bout of SIT. This single bout consisted of 4 × 30-sec maximal efforts on a stationary cycle ergometer (Monark Ergomedic 874 E, Monark, Sweden) against a resistance equivalent to 0.075 kg per kg body mass, as previously described (Richards et al. 2010a). Each 30-sec bout was separated by 4 min. Performance parameters during each training session (e.g., peak work rate, mean work rate, etc.) were measured/computed online via a hard-wire connection between the cycle ergometer and a personal computer, and using task-specific software (SMI Power 5.2.8, Delray Beach, FL). Twenty-three hours following completion of this single bout of SIT, RMR was redetermined.

Prior to each measurement of RMR, an intravenous catheter was inserted into an antecubital vein for subsequent blood sampling. Immediately following each measurement of RMR, venous blood (∼10 mL preserved with K3 ethylenediaminetetraacetic acid, plus ∼5 mL preserved with ethylene glycol tetraacetic acid/glutathione) was collected in chilled tubes, placed immediately on ice and centrifuged within 60 min of collection to isolate plasma. Circulating glucose concentration was analyzed immediately (from ∼1 mL whole blood) using an automated device (2300 STAT Plus Glucose Lactate Analyzer; YSI Inc., Yellow Springs, OH). Plasma samples were stored at −80°C until analysis. Enzyme-linked immunosorbent assays (ELISA) were used to measure, in duplicate, plasma concentrations of insulin (ALPCO Diagnostics, Salem, NH), leptin (Millipore Corporation, Billerica, MA), nonesterified fatty acids (Wako Diagnostics, Richmond, VA), and free and bound triiodothyronine (Bio-Quant, San Diego, CA).

### Study 2 – influence of SIT on total daily energy expenditure and fat oxidation

Following screening, research participants (*n* = 12) reported to the laboratory for the measurement of RMR (as described for study 1) and to complete a food preference survey. These data were used to create a controlled diet, designed to provide a consistent macronutrient intake for 3 days prior to the experimental protocol. Total free-living caloric needs were estimated as RMR × 1.5, and macronutrient distribution was 55% carbohydrate, 30% fat, and 15% protein. Fat content comprised 12–15% mono-unsaturated fat (as a percent of total calories), 6–9% polyunsaturated, and no more than 6% saturated fat. Research participants consumed only the food provided by the dietitians, however, two optional food modules (∼840 kJ each: 55% carbohydrate, 30% fat and 15% protein) were provided in the event of hunger. Research participants maintained their normal daily routines, but refrained from any structured exercise bouts during the 3-day lead-in to the study.

Total daily energy expenditure was determined using a whole-room indirect calorimeter, as previously described (Jung et al. 2011; Bergouignan et al. 2012), on two consecutive days, one of which began with a single bout of SIT (random order). The exercise and control days were completed in random order, with the single bout of SIT beginning at 09:00 on the exercise day. This bout was identical to that described in study 1, except this bout consisted of five (not four) 30-sec maximal efforts on a stationary cycle ergometer. On the control day, research participants sat quietly during this same time period. Research participants reported to the inpatient unit of the Clinical and Translational Research Center (CTRC) on day 1 at 07:00 and entered the calorimeter at 08:00, where they remained until 07:00 the next morning (day 2) when they were given the opportunity to shower. At 08:00 (day 2) research participants reentered the calorimeter, and remained there until 07:00 the following day (day 3). Napping or exercise and/or physical activity other than that prescribed by the investigators were not permitted. The data from each of the 2 days were extrapolated from their respective 23-h measurement periods to report 24-h values. Urine samples of 24 h were obtained and analyzed for urine nitrogen, urea nitrogen, and creatinine. These measures, along with measured VO_2_ and VCO_2_, were used to calculate the oxidation of carbohydrate, fat, and protein. In addition, 24-h urinary norepinephrine and epinephrine concentrations were determined via high-performance liquid chromatography and normalized to creatinine (Weinkove 1991).

During the stay in the whole-room indirect calorimeter, to accommodate the decrease in energy expenditure compared with usual free-living energy expenditure, dietary intake was modified such that total calories were equal to RMR × 1.4. The macronutrient distribution remained unaltered from the precalorimeter diet. Meals were provided at 10:00, 14:00, and 20:00; the distribution of calories was 33.3% at each meal.

### Statistical analysis

These were controlled, repeated measures studies. Accordingly, in study 1 the influence of a single bout of SIT on RMR was examined via one-way, repeated measures analysis of variance (ANOVA). Typical error was calculated using established procedures as previously described (Hopkins 2000). Similarly, in study 2 the influence of a single bout of SIT on total daily energy expenditure and fat oxidation was also examined via one-way repeated measures ANOVA. Multiple comparisons of factor means were performed using the Newman–Keuls test. The level of statistical significance was set at *P* < 0.05. Data are expressed as mean ± SE.

## Results

### Study 1 – influence of SIT on RMR

Fifteen research participants were prescribed four sprints each. From this total of 60, 58 sprints (∼97%) were successfully completed. Two research participants were unable to complete the final (4th) sprint of their session due to nausea and light-headedness. Performance parameters during the single bout of SIT were comparable to previous studies and typical of healthy young men. Peak power outputs during the first sprints ranged from 487 to 955 W (mean ± SE: 738 ± 36 W), and were decreased (*P* < 0.001) during the final sprints (497 ± 72 W). Similarly, mean power outputs were decreased (*P* = 0.004) in the final sprints compared with first (547 ± 43 vs. 385 ± 46 W).

The RMR measurement (Table 2) was highly reproducible across the three evaluations not preceded by a single bout of SIT (typical error: 2.5%). A single bout of SIT did not increase RMR measured 23 h later (*P* = 0.12). Similarly, resting respiratory exchange ratio (Table 2) was unaffected (*P* = 0.91) by a single bout of SIT. Body mass was also unaffected by a single bout of SIT (*P* = 0.74; data not shown). Consistent with the lack of influence of a single bout of SIT on RMR and respiratory exchange ratio, SIT did not affect circulating fasting concentrations of glucose, insulin, nonesterified fatty acids, leptin, and free and total triiodothyronine measured the following day (Table 2; all *P* > 0.05).

**Table 2:** Metabolic and thermogenic/metabolic circulating factors prior to and 23 h following a single bout of sprint interval training (SIT).

	B1	B2	B3	Post-SIT
RMR (kJ/day)	7147 ± 222	7149 ± 246	6987 ± 245	7195 ± 285
RER	0.86 ± 0.01	0.86 ± 0.01	0.85 ± 0.01	0.85 ± 0.01
Glucose (mmol/L)	5.00 ± 0.11	5.04 ± 0.13	5.04 ± 0.10	4.98 ± 0.12
Insulin (pmol/L)	24.4 ± 4.5	25.4 ± 3.7	22.9 ± 3.5	23.9 ± 3.2
NEFA (mmol/L)	0.31 ± 0.04	0.34 ± 0.04	0.36 ± 0.04	0.34 ± 0.04
Leptin (ng/mL)	7.27 ± 0.82	7.28 ± 0.90	7.31 ± 1.08	7.85 ± 1.10
Free T3 (pmol/L)	3.00 ± 0.14	3.05 ± 0.13	3.02 ± 0.17	2.96 ± 0.13
Total T3 (nmol/L)	1.05 ± 0.09	1.08 ± 0.09	1.01 ± 0.09	0.98 ± 0.10

Data are mean ± SE. All variables were determined on four separate occasions. Measurements were performed over two pairs of consecutive mornings; each pair was separated by 7 days. Immediately following either the first or third data collection (randomly assigned) research participants completed a single bout of SIT. B1, B2, and B3 refer to baseline values determined from measurements not preceded by SIT. RMR, resting metabolic rate (1 kcal = 4.1868 kJ); RER, respiratory exchange ratio; NEFA, nonesterified fatty acids; T3, triiodothyronine. All comparisons are *P* > 0.05.

### Study 2 – influence of SIT on total daily energy expenditure

Twelve research participants were prescribed five sprints each. All sprints (100%) were successfully completed, although one research participant required additional recovery time between sprints (1–2.5 extra minutes of recovery after completing sprints 2, 3, and 4) on account of sensations of nausea and light-headedness. Performance parameters during the single bout of SIT were comparable to those reported in study 1. Peak power outputs during the first sprints ranged from 597 to 1142 W (805 ± 58 W), and 582 to 983 W (723 ± 46 W) during the final sprints. Mean power outputs were decreased (*P* = 0.004) in the final sprints compared with first (560 ± 25 vs. 434 ± 26 W).

**Figure 1:**
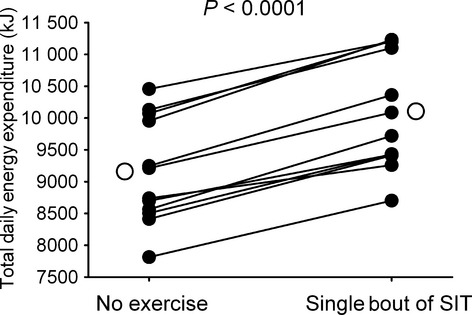
A single bout of sprint interval training (SIT) increased total daily energy expenditure in all research participants. Closed circles and solid lines depict individual research participant responses. Open circles represent mean values.

**Figure 2:**
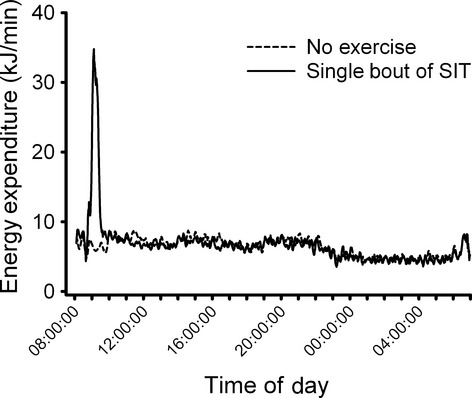
Minute-by-minute energy expenditure during a sedentary day and a day beginning with a single bout of sprint interval training (SIT). Data are mean values.

A single bout of SIT increased total daily energy expenditure in every subject (Fig. 1; *P* < 0.001). The magnitude of increase was 946 ± 62 kJ/day, reflecting a mean increase in TDEE of approximately 10%. Visual inspection of the mean minute-by-minute data (Fig. 2) suggested that the SIT-induced increase in total daily energy expenditure could be attributed to the period of time during, and immediately following SIT. In order to investigate this further, the minute-by-minute data for each subject were grouped into intervals of 4-h (Table 3). Compared to the same periods on the control day, SIT increased energy expenditure between 08:00 and 12:00 noon only (*P* < 0.001).

**Table 3:** Energy expenditure and respiratory exchange ratio during a 24-h period on a sedentary day and a day beginning with a single session of sprint interval training (SIT).

Time of day	Energy expenditure (kJ/min)		Respiratory exchange ratio	
	
	Sedentary	SIT	Sedentary	SIT
08:00–11:59	7.05 ± 0.22	10.26 ± 0.86*	0.81 ± 0.01	0.91 ± 0.01*
12:00–15:59	7.25 ± 0.20	6.9 ± 0.43	0.84 ± 0.01	0.86 ± 0.01
16:00–19:59	6.9 ± 0.19	6.60 ± 0.35	0.86 ± 0.01	0.85 ± 0.01
20:00–23.59	6.55 ± 0.19	6.11 ± 0.27	0.86 ± 0.01	0.84 ± 0.01
00:00–03.59	4.82 ± 0.16	4.43 ± 0.40	0.85 ± 0.01	0.84 ± 0.01
04:00–07:00	5.32 ± 0.23	5.01 ± 0.41	0.84 ± 0.01	0.84 ± 0.01

Data are mean ± SE. 1 kcal = 4.1868 kJ.

* Denotes difference between sedentary and SIT for corresponding time period (*P* < 0.001). All other comparisons are *P* > 0.08.

**Figure 3:**
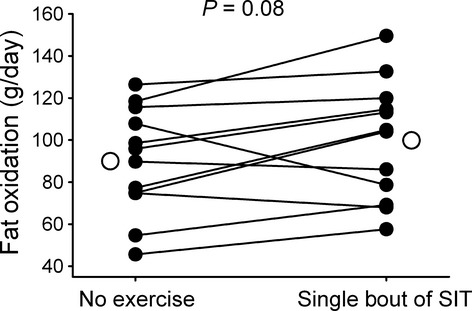
A single bout of sprint interval training (SIT) increased fat oxidation in 10 of 12 research participants, but this increase did not attain statistical significance. Closed circles and solid lines depict individual research participant responses. Open circles represent mean values.

**Figure 4:**
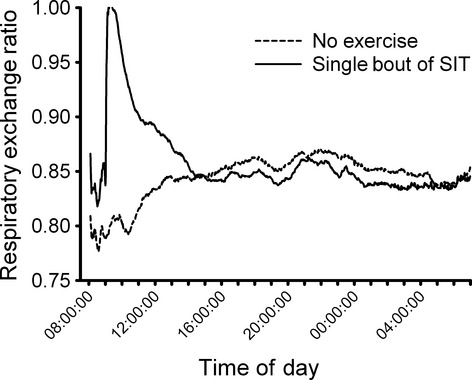
Minute-by-minute respiratory exchange ratio during a sedentary day and a day beginning with a single bout of sprint interval training (SIT). Data are mean values.

To further explore the influence of SIT on 24-h macronutrient metabolism, we were able to use respiratory and urine-derived data to quantify changes in substrate oxidation. Based on respiratory exchange ratio and urine/urea nitrogen data, total daily fat oxidation was increased in 10 of 12 subjects (Fig. 3); however, this increase did not attain statistical significance (*P* = 0.08). Minute-by-minute (Fig. 4) and 4-hourly (Table 3) respiratory exchange ratio values were increased between 08:00 and noon only (*P* < 0.001). Data pertaining to macronutrient balance (intake and expenditure) are presented in Table 4. In accordance with our experimental design and goals, energy balance was not maintained during the day that included the single bout of SIT. The differences in macronutrient oxidation between the sedentary and sprinting day did not attain statistical difference (*P*-values ranged between 0.07 and 0.70). Although the overall energy expenditure response to sprint exercise was consistent across all subjects, the macronutrient oxidation responses were much more variable.

**Table 4:** Substrate intake and oxidation during a 24-h period on a sedentary day and a day beginning with a single session of sprint interval training (SIT).

	Sedentary	SIT
Energy balance (kJ/day)*	662 ± 184	−326 ± 183
Energy expenditure (kJ/day)*	9169 ± 243	10,111 ± 260
Energy intake (kJ/day)	9828 ± 287	9787 ± 304
Protein balance (g/day)	12.4 ± 4.4	13.3 ± 4.1
Protein oxidation (g/day)	78.2 ± 3.2	67.4 ± 3.5
Protein intake (g/day)	90.5 ± 2.7	89.7 ± 2.7
Carbohydrate balance (g/day)	65.5 ± 16.8	38.5 ± 12.8
Carbohydrate oxidation (g/day)	240.2 ± 19.8	265.8 ± 14.5
Total carbohydrate intake (g/day)	331.2 ± 10.0	330.5 ± 10.0
Fiber carbohydrate intake (g/day)	25.5 ± 2.4	26.3 ± 1.3
Fat balance (g/day)	−9.7 ± 7.9	−20.6 ± 8.2
Fat oxidation (g/day)	90.0 ± 7.3	99.9 ± 8.1
Fat intake (g/day)	80.2 ± 2.4	79.3 ± 2.7

Data are mean ± SE. 1 kcal = 4.1868 kJ.

* Denotes difference between sedentary and SIT day (*P* < 0.001). All other comparisons are *P* > 0.05.

Additional analysis of urine revealed no appreciable influence of SIT on catecholamines. Neither epinephrine (control = 4 ± 1 vs. SIT: 5 ± 1 μg/g creatinine; *P* = 0.16) nor norepinephrine concentration (Sedentary: 19 ± 3 vs. SIT: 20 ± 3 μg/g creatinine; *P* = 0.33, *n* = 10) was affected by SIT.

## Discussion

Assuming no change in dietary intake, and based on metabolic efficiency and the caloric cost of weight gain, an increase in total daily energy expenditure of ∼200–600 kJ (∼50–150 kcal) has been estimated as being sufficient to prevent weight gain in ∼90% of adults living in industrialized countries (Hill et al. 2003; Stroebele et al. 2011). The purpose of these studies was to determine if a single bout of SIT could evoke an increase in RMR and/or total daily energy expenditure of at least 200–600 kJ. We report that a single bout of SIT does not influence resting metabolism, determined ∼23 h post sprinting, but it does increase total daily energy expenditure by ∼950 kJ.

In our first study, RMR was measured on four separate occasions, over two pairs of consecutive mornings; each pair separated by 1 week. Immediately following either the first or third RMR measurement, a single bout of SIT was completed. This first study represents a logical extension of our previous report that RMR was unaffected by either short term (2 weeks) or a single bout of SIT (Richards et al. 2010a); although the conclusions reached in this study are consistent with our older data, the original protocol was not designed such that it would address this specific research question with sufficient scientific rigor to reach a definitive conclusion. The present design provided the opportunity for a thorough determination of the reliability of the RMR measurement, and increased the confidence with which the influence of a single bout of SIT on RMR could be reported. The measurement of RMR was highly reproducible (typical error: 2.5%), and compares favorably with previously reported indices of reliability (Branson and Johannigman 2004; Holdy 2004). Furthermore, in this study, additional mechanistic insight is provided via the examination of circulating factors known to be of thermogenic/metabolic importance (glucose, insulin, nonesterified fatty acids, triiodothyronine, and leptin). Based on our data, and in light of the significant exertion associated with SIT, together with the established positive association between exercise intensity and magnitude of increase in metabolic rate during recovery (Gore and Withers 1990; Borsheim and Bahr 2003), we surmised that the duration of the metabolic recovery from SIT must be markedly less than 23 h. Consequently, our second study was undertaken to directly determine the influence of a single bout of SIT on 24-h energy expenditure, measured continuously in a whole-room calorimeter for more than 20 h following exercise. Noteworthy, in light of the lack of effect of four sprints on RMR, we wished to increase the magnitude of the perturbation. Most sprint protocols involve between four and eight 30-sec sprints; increasing the number of sprints from four to five seemed a conservative way to increase the “stimulation” while preserving the achievability of the intervention.

A single bout of SIT increased total daily energy expenditure by ∼950 kJ/day (225 kcal/day; ∼10%). Closer inspection of the data revealed that this increase could be attributed almost entirely to the thermogenic cost of the actual exercise, and the elevation of metabolic rate during the 2–3 h immediately following its completion. Our observations are supported by several recent reports describing the energy expenditure (determined intermittently) during and following a single bout of SIT (Burns et al. 2012; Matsuo et al. 2012; Chan and Burns 2013; Freese et al. 2013), but contradict one study that reported higher oxygen consumption (relative to a sedentary control condition) 8 h following sprint exercise (Hazell et al. 2012). In agreement with our data, oxygen consumption returned to basal values 24 h after sprinting. One important difference between this and the present study is the protocol and method of quantifying energy expenditure. In the previous study, research participants were allowed to leave the laboratory after exercising, and return at predetermined intervals for indirect calorimetry. It is possible, and a limitation acknowledged by the authors, that despite rigorous dietary controls and clear instructions provided to the research participants as to the permitted physical activities performed outside the laboratory, the lack of total control may have contributed to the prolonged recovery from sprinting. In this regard, we believe that our protocol utilizing a whole-room calorimeter was superior, although the decreased external validity resulting from constrained free-living behaviors is recognized.

The increased metabolic rate following exercise (EPOC: excess postexercise oxygen consumption) has been studied extensively; readers are referred to several comprehensive reviews for detailed discussion beyond the scope of the present manuscript (Borsheim and Bahr 2003; Speakman and Selman 2003; LaForgia et al. 2006). The intensity of exercise appears to have a greater regulatory role on EPOC than the duration, and potential physiological determinants of EPOC include restoration of acid-base balance, time course of the return to baseline values of cardiovascular and pulmonary function, elevated body temperature, replenishment of adenosine triphosphate and other components of the phosphagen system, and thermogenic effects of circulating endocrine factors such as catecholamines and thyroid hormones. With regard to the latter, we have shown that although a single bout of SIT increased total daily energy expenditure, it did not affect 24-h urinary catecholamine concentration, and plasma concentrations of triiodothyronine and leptin. In addition, insulin, glucose, and nonesterified fatty acids all returned to baseline within 23 h of sprinting. Furthermore, while some have suggested that the duration and contribution of EPOC to total daily energy expenditure has been exaggerated, and the thermogenic contribution of the actual exercise bout to 24-h energy expenditure is more relevant than EPOC (Borsheim and Bahr 2003; Speakman and Selman 2003; LaForgia et al. 2006); recent aerobic exercise studies suggest otherwise (Knab et al. 2011). In our study, the thermogenic effects of a single bout of SIT were brief (∼4 h). This discrepancy is likely due to differences in exercise intensity, duration and total work done. Alternatively, the elevation in energy expenditure in the resting/recovering state may have been dampened by a fatigue-induced decrease in physical activity that in turn contributed to the relatively brief overall thermogenic effect. In this regard, use of accelerometer/radar to quantify physical activity within the chamber may have been useful; however, we did not collect these data. Activity within the chamber was carefully controlled. Eating and sleeping times were standardized, and napping or exercise and/or physical activity other than that prescribed by the investigators were not permitted. Thus, we did not feel that the use of radar/accelerometers was justified.

Our rationale for not maintaining energy balance in the current study was based on the proposal that in the absence of a compensatory increase in dietary intake, a daily increase in 24-h energy expenditure approximating 200–600 kJ (∼50–150 kcal) is sufficient to prevent weight gain (Hill et al. 2003; Stroebele et al. 2011). While a single bout of SIT does not affect individual macronutrient oxidation per se, it does increase total daily energy expenditure by ∼950 kJ. Thus, in addition to being a potential strategy for weight maintenance, regular SIT may prove to have some utility for weight loss. In support of this, favorable modification of body composition following short-term (3–12 weeks) SIT has been reported (Macpherson et al. 2011; Heydari et al. 2012).

From a public health perspective, there are several important points to consider before widespread recommendation and support for SIT. Frequently described as a time-efficient alternative to traditional endurance exercise, it is worth noting that although the time spent sprinting is relatively short (e.g., 2.0–2.5 min in the current protocol), incorporation of a warm-up, cool-down, and recovery periods between sprints increases the duration of a single SIT session to ∼30 min. Furthermore, although SIT has been rated more enjoyable than traditional endurance exercise (Bartlett et al. 2011), it seems unlikely most people (noncompetitive athletes) would be able to sustain the high degree of intrinsic motivation required to repeatedly reproduce the significant exertion associated with SIT. Perhaps a more conservative approach would be to consider incorporating SIT into a regular program of activity, rather than using it to replace all traditional endurance exercise. Alternatively, given the rapid time course in which SIT is able to evoke physiological adaptations (Burgomaster et al. 2005; Gibala et al. 2006; Richards et al. 2010a), SIT may prove particularly effective during the early initiation of a new program of exercise (e.g., as a gateway exercise), or from a sport performance perspective, as a strategy to promote swift returns to fitness following the off-season.

In summary, the purpose of these studies was to determine if a single bout of SIT could evoke an increase in RMR and/or an increase in total daily energy expenditure of at least 200–600 kJ. We report that a single bout of SIT does not influence resting metabolism, determined 23 h later, but it does increase total daily energy expenditure by ∼950 kJ. These data imply that a daily single bout of sprint interval training may be sufficient to prevent weight gain in ∼90% of adults living in industrialized countries, and suggest that the vast majority of the thermogenic response to SIT is due to the actual exercise and the brief period of recovery following its completion.
